# Infants Are More Likely Than Older Children to Have Surgery for Cervical Infections

**DOI:** 10.1155/2018/7824380

**Published:** 2018-05-28

**Authors:** Jonathan A. Harounian, Andrew R. Azab, Christopher A. Roberts, Michele M. Carr

**Affiliations:** ^1^Department of Otolaryngology-Head and Neck Surgery, Temple University, Philadelphia PA, USA; ^2^Penn State College of Medicine, Hershey PA, USA; ^3^Department of Otolaryngology-Head and Neck Surgery, West Virginia University, Morgantown WV, USA

## Abstract

**Objectives:**

To identify differences in cervical infection management in infants versus older children.

**Methods:**

Charts of patients 0–18 years, diagnosed with a cervical infection at our institution between 2004 and 2015, were included. Age, gender, presenting symptoms, comorbidities, CT scan findings and management including admission, procedures, antibiotics, cultures, length of stay, readmission rates, and complications were included.

**Results:**

239 patients were included: mean age was 4.6 years, with 55.6% boys and 44.4% girls. Mean length of stay was 3.2 days, with no significant difference between age categories. 12.55% were readmitted within 30 days with no significant difference when stratified for age (p = 0.268). The most common presenting symptoms were fever (74.3%), swelling (71.4%), and neck pain (48.2%). Infants had fewer symptoms documented than older children. 51% has lateral neck infections, and these were more common in younger children (p < 0.001). The most common antibiotic used was amoxicillin-clavulanic acid in 53.96% of inpatients and 48.05% of outpatients. Infants were most likely to have MRSA isolates (29.2% versus 11.7% of older children, p = 0.011). 70.0% went to the operating room for incision and drainage procedures. Younger children were more likely to undergo surgery, with an odds ratio of 2.38 for children under 1 year. (p = 0.029). 90.9% of infants underwent surgery with radiolucencies of at least 1 cm diameter in contrast to 50% of children over 8 years old.

**Conclusions:**

This study emphasizes the importance of considering early operative treatment of cervical abscesses in infants despite fewer symptoms and smaller radiolucencies on CT.

## 1. Introduction

The diagnosis and management of neck infections are an ongoing challenge for clinicians [1, 2]. It is often difficult to make a diagnosis of abscess versus cellulitis and it is difficult to know when surgical intervention is appropriate.

Evaluation of neck infections first involves assessment of airway patency by direct examination of the oropharynx, lateral neck radiographs, and computed tomography (CT) with contrast. Freling et al. assessed the predictive value of using CT scanning in the diagnosis of cervical abscesses and found that, in their hands, the positive predictive value was 82% and negative predictive value 100%, with presence of air indicating abscess in all cases [3]. Most neck infections are in the pharyngeal regions or in the lateral neck. Age differences in location have been reported, with retropharyngeal infections being most common in children between 2 and 4 years old [4] although they are also found in other age groups, including infants [2]. Laboratory studies including complete blood count and blood culture may be helpful in management as well [5].

Neck abscesses are often polymicrobial, with an increasing incidence of methicillin-resistant* Staphylococcus aureus* (MRSA) isolates more recently [6]. Rarer etiologies include* Bartonella henselae* and atypical mycobacterial infections [7]. Complications of retropharyngeal infections include septicemia, aspiration pneumonia, jugular vein thrombosis, and carotid artery rupture; relapse occurs in 5% of patients [8, 9].

Current treatment is generally based on CT findings and extent of airway obstruction. Airway compromise is managed by intubation, immediate surgical drainage, and early empiric antibiotic therapy [10]. In cases without airway obstruction, there is no consensus on appropriate management [11]. Average length of hospital stay for children with cervical abscesses without mechanical ventilation is 3–5 days [5]. Studies have examined the size of CT radiolucencies in deep neck abscesses and the frequency of eventual surgical drainage [12, 13]. Larger abscesses with larger radiolucencies are more likely to be treated surgically, with cutoff size in the 2 to 2.5 cm range [13].

There is a large degree of variability in pediatric cervical abscesses based on age, specific comorbidities, location of abscess, how the disease progresses, bacterial etiology, and response to antibiotics or drainage. Our hypothesis was that there were age related differences in cervical abscess presentation and management.

## 2. Materials and Methods

A retrospective review was performed on all patients between the ages of 0 and 18 years that were admitted and diagnosed with a neck abscess at Penn State Hershey Medical Center between January 1st, 2004 and May 12th, 2015. Children who were not diagnosed with a neck abscess by the admission team by the time of discharge and children who had incomplete records were excluded. Admission teams included all pediatrics, pediatric surgery, otolaryngology, and plastic surgery. We used CPT codes for incision and drainage of neck abscess, 21501 (incision and drainage, deep abscess or hematoma, soft tissues of neck, or thorax), 42720 (incision and drainage abscess, retropharyngeal or parapharyngeal, and intraoral approach) and 42725 (incision and drainage abscess; retropharyngeal or parapharyngeal, and external approach), and ICD-9 codes for cellulitis and abscess of neck (682.1) and retropharyngeal abscess (478.24) to obtain the patient population. Data was then filtered for exclusion criteria, and relevant data was extracted from each patient chart. Age, gender, presenting symptoms, comorbidities, and CT and other imaging findings were reviewed. Management including level of admission, procedures, antibiotics, bacterial cultures, readmission rates, length of stay, and complications were included.

Data analysis was done using SPSS 22 (IBM Corp. Released 2013. IBM SPSS Statistics for Windows, Version 22.0. Armonk, NY: IBM Corp).

This study was approved by the Institutional Review Board, #2633.

## 3. Results

**Figure 1:**
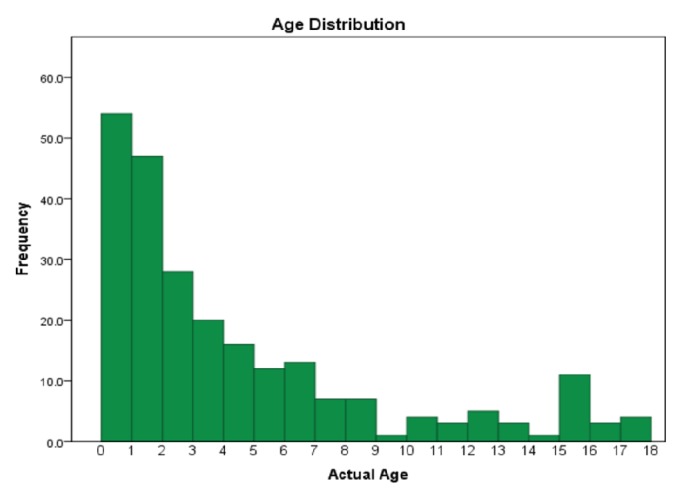
Age distribution for all children diagnosed with neck infection.

Four hundred and twenty-two charts were reviewed of which 239 were patients who met the inclusion criteria and were diagnosed with a neck abscess. These were included in our study. The remaining 183 patients were excluded due to incomplete records, incorrect site of infection (i.e., thorax, etc.), or miscoding; there was no mention of cervical infection in the charts of the latter 2 groups. Notes within 6 months of the date of coding were reviewed to determine exclusion. Fifty-four (22.6%) were less than 1 year of age, 95 (39.7%) were 1–3 years old, 48 (20.1%) were 4–7 years old, and 42 (17.6%) were 8–18 years old (Figure 1). The mean age was 4.60 years (range: 0.11-17.83). In more recent years, the percentage of patients under 1 year of age was significantly higher, ranging from a low of 5.3% in 2006 to a high of 45.5% in 2015 (p = 0.067).

The mean BMI was 18.38 (range: 16.84-44.10). One hundred thirty-three (55.6%) of the patients were male and 106 (44.5%) of the patients were female. The majority of patients were Caucasian (67.6%) followed by African American (12.2%), 7.4% were Hispanic and less than 1% were Asian, 2.9% were mixed race, and the remainder were not identified.

196 (82.0%) patients were admitted to hospital. The remaining 18% required a hospital stay of less than 24 hours and, thus, were not included in the number of reported admissions. The mean length of stay was 3.2 days (range: 0.05-54.63). Length of stay was not significantly different between patients of each age group (p = 0.630), reported in Table 1. Thirty-one (12.6%) patients were readmitted within 30 days. The 30-day readmission rate was equivalent for all age groups (p = 0.268). The mean length of stay for readmission was 2.1 days (range: 0.00-7.00), again with no significant difference between age groups, but the groups were small. 173 (70.0%) of the patients went to the operating room (OR) for incision and drainage. Of the patients that underwent operative intervention, 68.2% were younger than 3 years old and 13.3% were older than 8 years (p = 0.003). There was a significant difference between the ages of those undergoing surgery, with younger children requiring surgery more often (p = 0.004), which is shown in Table 2.

Presenting symptoms by age are seen in Table 3. Analysis of symptom presentation showed that fever (74.3%), swelling (71.4%), and neck pain were most common (48.2%). These are percentages of positive findings for the whole group; in many cases there was no mention of symptoms in the medical record. Table 3 lists presence of symptoms only for cases where the symptom was recorded in the chart, excluding those where there was no mention. This table shows that there were significant differences in the frequency of neck pain, neck stiffness, sore throat, vomiting, and hoarseness, with all of these being least common in infants.

The most common abscess location was lateral cervical (51.0%), with retropharyngeal being the second most common (Table 4). There were age differences in location, with lateral cervical abscesses being most common in infants. Retropharyngeal abscesses became more frequent with increasing age. Cervical abscesses comprised the largest group undergoing surgery (42.9%), followed by retropharyngeal (14.6%) (p = 0.001).

Intravenous (IV) antibiotic orders were found in the charts of 204 patients (82.6%). The most common IV antibiotic used was ampicillin/sulbactam (53.96%) followed by clindamycin (19.1%) and a combination of antibiotics (16.2%). Oral antibiotics were used in 231 patients at some point. The most common oral antibiotic used was amoxicillin and clavulanic acid (48.1%) followed by clindamycin (29.9%). 70 patients did not go to the OR; of these 6 (2.4%) patients had a CT guided aspiration and 3 (1.2%) patients had ultrasound (US) guided aspiration. One (0.4%) patient had an incision and drainage in the OR and US guided aspiration.

179 patients had a mean white blood cell count of 19.12 thousand per microliter (range: 5.09-55.00) with no significant difference between age groups or groups based on whether operative intervention was undertaken. 176 (71.3%) patients had their abscesses cultured. The most common culture results were no growth (24.4%),* Staphylococcus aureus* (23.9%), MRSA (16.5%), Beta* Streptococcu*s group A (12.5%), and* Streptococcus viridans* (10.2%). There were significant age differences for culture results (p = 0.011). Children aged 1 and under were most likely to have* S. aureus* and MRSA; this accounted for 70.9% of their results, with 14.6% having no growth. In older children, culture results were more variable and were frequently mixed. Group A Strep was most common (31.0%) in the 4–7 year old group,* S. aureus* (22.2%), and* S. viridans* (14.8%) and Group A Strep (11.1%) in the children aged 8 to 18 years.

A CT of the neck was performed on 195 (81.6%) patients. CT results are listed in Table 5. Rim enhancing radiolucencies were more common in children under 7 years old (30.0% had them) than in those who were older (11.4%, p = 0.043). 167 patients had a CT radiolucency diameter recorded, with a mean diameter of 2.08 ± 1.48 centimeters (cm) (range: 0.10–8.00). There was no significant difference in the size of the radiolucency between age groups. Most children who were taken to the OR had a radiolucency of 1 cm or more (96.7%). However, only 76.3% of children with radiolucencies of 1 cm or more eventually underwent operative intervention. One cm radiolucencies were present in 90.9% of infants who were managed surgically, but only 83.1% of children aged 1–3 years, 67.6% of children aged 4–7 years, and 50.0% of children aged 8–18 years who underwent surgery. One cm radiolucencies in older children were more likely to be managed nonoperatively.

Multiple logistic regression revealed that significant contributors to whether children underwent surgery were age, location of the abscess, and radiolucency size (P < 0.001); odds ratios with confidence intervals are listed in Table 6. Infants, patients with lateral neck abscesses, and patients with CT radiolucencies of at least 1 cm were more likely to undergo surgery.

Thirty-two (13.4%) patients presented with comorbidities, mainly asthma (7 patients). Only 10 (4.5%) patients experienced complications, including jugular vein thrombosis, a second surgery, intravenous infiltration, and laryngospasm requiring reintubation. Of the 239 patients, only 2 (0.8%) went to the Pediatric ICU. Both were children under 3 years old with MRSA infections.

## 4. Discussion

This group of children diagnosed with cervical abscess by the time of hospital discharge was managed by a variety of clinicians from a variety of specialties and often with collaboration of specialty teams. There was no specific protocol in place in this academic tertiary care hospital to diagnose or treat children with cervical infections. At that time, literature was suggesting that some children with cervical abscesses as determined by CT could be managed without surgical drainage [12, 13]. Our goal in reviewing these charts was to monitor our outcomes in general, in order to improve care. We suspected that clinical courses were different depending on the age of the child—infants were more different than older children.

Sixty-one percent of our group of children with neck infections were under 3 years old, which has not been seen in previous reports. In Cabrera et al., for example, 89 pediatric patients admitted for deep neck infections were equally distributed among all age groups [1]. Our mean length of stay was 3.18 days, which is shorter than in previous studies [5]. Previous studies also found no statistical difference between age groups in regard to length of stay [5], as was the case in our study. Readmission rates were high in our study (12.55%) as compared to a recent study in Wisconsin study (3.5%) [14]. Readmission did not vary significantly between age groups in the Wisconsin study [14] or in our group. Younger patients tended toward longer lengths of stay on readmission than older ones, although we did not show statistical significance in this small group.

Presenting symptoms found in our group correlate with a recent study of pediatric patients in Italy [3]. Fever, neck pain, and swelling were their most frequent symptoms, and we found this as well. In our group, infants had the fewest presenting symptoms, with neck swelling most common (95.9% among those patients for whom this symptom appeared in the medical record). Cmerjek et al. also found this in their group of 25 infants with neck abscesses as 92% had neck swelling as a presenting symptom [15]. Symptoms may not be as useful in this age group, so presence of neck swelling should instigate further investigation to rule out a neck abscess.

Seventy percent of our patients were treated in the operating room. Patients younger than 3 years old were more likely to undergo operative intervention than older patients. Infants undergoing surgery also had smaller CT radiolucencies than older patients. Combined with the fact that they have fewer presenting symptoms, infants may require earlier intervention than other age groups. Although there is controversy about the management of pediatric deep neck abscesses, it is multimodal and prompt recognition and treatment will reduce morbidity [4, 11, 16]. Knowing that infants present more subtly and end up having surgery with smaller CT radiolucencies is helpful in expediting their management.

A 1995-retrospective study of 117 children at the Children's Hospital of Pittsburgh showed that peritonsillar space infections were most common (49%) among neck infections [17]. Our study showed very few peritonsillar infections (2.4%), though this could be a result of not including the ICD-9 code for peritonsillar abscess. Their second most common location was retropharyngeal (22.0%), which was similar to our results. This study is still used as an epidemiological reference in current resources but may not represent current trends.

In the same Pittsburgh study, the most common pathogens infecting these pediatric patients were aerobes including beta-hemolytic* Streptococcus* (18%) and* Staphylococcus aureus* (18%) [17]. We found a higher incidence of* Staphylococcus aureus* (23.86%) and MRSA (16.48%) and a lower incidence of* Streptococcus*. The increased prevalence of MRSA contributes to changes in management of these patients [6].

Interestingly, a retrospective study by Novis et al. characterizing pediatric deep neck infections at the national level revealed a statistically significant decrease in the percentage of retropharyngeal abscess patients managed surgically (48% versus 38%) and in the average length of hospital stay (4.6 versus 3.9 days) between 2000 and 2009 [18]. This trend implies a shift in management strategies. A retrospective review of 39 patients conducted by Grisaru-Soen et al. concluded that many patients with retropharyngeal and parapharyngeal abscesses can be “treated successfully without surgery” [19]. In the study, CT scanning was performed on 37 patients, of whom 17 underwent surgical drainage. Thirteen children had pus at surgery; 12 had been suspected of having an abscess on their CT scan. All patients received IV antibiotics. There was no significant difference in the duration of hospital stay (mean was 8 days) between those who underwent surgery and those who were treated with antibiotics alone. There were no treatment failures and no complications in either of the two groups. In addition to reinforcing the concept that medical management alone may be an acceptable means of managing these abscesses, the findings also suggest that CT scans are useful in diagnosing and assessing the extent of infection, but they are not always accurate [19]. In our study, we saw more patients managed surgically (70.0%) but a shorter hospital stay (3.18 days) as compared to the previous study. Ideally, a noninvasive approach would be preferred, but this is not always the optimal course of treatment to reduce hospital stay.

Johnston et al. observed 32 pediatric patients treated with surgery (10 patients) and a 24-48 hour trial of IV antibiotics (22 patients) [20]. Of the latter group, thirteen children failed antibiotic therapy and were drained surgically, comprising the late surgical intervention group. This study revealed that patients with early surgical intervention (defined as those patients who underwent surgery less than 24 hours after admission) had a shorter average hospital stay (2.7 days) than an IV antibiotic therapy group (3.3 days). The length of stay between these groups was not statistically significantly different. Patients who failed medical therapy also had significantly larger abscess diameters than those who responded to IV antibiotic therapy. The authors conclude that a trial of IV antibiotics does not adversely affect outcome and may negate the need for surgery, especially in patients with smaller abscesses. Although there is a limited cohort in this study, it shows the complicated multifactorial nature of this disease. Characterization of severity may be the next step in improving pediatric cervical abscess management.

There is some evidence in the literature suggesting that younger children may be more likely to be managed surgically. For retropharyngeal and parapharyngeal abscesses, Grisau-Soen found a significant difference in the age of patients who underwent surgery versus those treated with antibiotics alone (2.5 versus 5.6 years) [19]. Wong et al. found that the mean age of patients with abscesses smaller than 2.5 cm in diameter on CT that required drainage was 3.66 years versus 5.72 years for patients responding to antibiotics [13]. There was no significant difference in length of hospital stay [13]. Duggal et al. found that infants under 16 months had a higher frequency of MRSA in culture results from cervical abscesses and 80% of MRSA isolates came from lateral neck abscesses [21]. This is similar to our group, with 29.2% of our infants having MRSA isolates (compared to 11.7% of children 1 year old and older) and 31.4% of lateral neck abscesses in infants growing MRSA. We found that methicillin sensitive S. aureus was slightly more common in lateral neck abscesses, especially in infants (22% over all age groups, 45% in infants).

It may be that infants are more likely to come to surgery because they typically have easily accessible abscesses (versus a retropharyngeal location) and are more likely to have MRSA and thus not respond to antibiotics.

Previous studies have correlated size of CT radiolucencies in neck infections with the need for surgical drainage. These have concentrated on retropharyngeal and parapharyngeal abscesses. McClay et al. determined the effectiveness of using IV antibiotics alone to treat clinically stable children with clearly defined deep neck abscesses diagnosed by CT with at least 1 cm radiolucencies. Ninety-one percent (10/11) of the children responded to IV antibiotics alone, all of whom improved clinically by 48 hours and had no complications [12]. Wong et al. found that 85% of retropharyngeal and parapharyngeal abscesses larger than 2.5 cm were drained, and for these larger lesions, the mean age between those undergoing surgery and those responding to antibiotics was not significantly different [13]. In our study, most patients had lateral abscesses, but Wong et al. prompted our examination of radiolucency size as a predictor for operative intervention. Our data show that 90.9% of infants with radiolucencies of at least 1 cm undergo operative intervention, suggesting that a guideline based on CT findings needs to be altered depending on the age of the child. An infant with a 1 cm radiolucency is almost 3 times more likely to have surgical drainage than a child older than 1 year with the same CT findings. This is important for clinicians following an algorithm that states that radiolucencies should be larger than 2 cm in diameter before surgical intervention is considered.

There are limitations to our study. This is not a topic that lends itself to a randomized controlled trial, so a retrospective review is the only way we can learn about it. However, the medical record may be incomplete or erroneous and patients may have received some care in other facilities or did not follow up with our facility. Also, CPT and ICD-9 codes were used to generate our patient list. These codes yielded many patients who did not have neck infections. Other patients of interest were likely missed because of miscoding. Patients were managed by different specialty teams and attending physicians, so workup varied. Decisions were made based on the team's clinical judgment, not a predetermined uniform management algorithm. Diagnosis of abscess may have been erroneous in patients who did not undergo surgery. Also, this study spanned 10 years so changes in bacterial antibiotic resistance and changes in medical protocols are possible. This study represents a single institution and findings may not be generalizable to other populations.

## 5. Conclusion

 Our study shows that neck infections are presenting in younger children more than in the past. Infants have fewer classic neck abscess symptoms but are more likely to have MRSA infections and lateral neck infections and to undergo operative intervention. CT findings are less remarkable in this group, so this alone cannot be used as a guideline to decide whether a child should be treated operatively. Emergency room physicians today will see infants in this clinical context and should know that infants are more likely to undergo surgical drainage than older children despite their more subtle presentation.

## Figures and Tables

**Table 1:** Analysis of length of stay (days) among pediatric age groups.

**Length of Stay for First Admission for Neck Abscess Patients**				
**Age ** **(years)**	**N**	**Mean**	**Lower 95**%** CL for Mean**	**Upper 95**%** CL for Mean**
<1	54	3.42	2.43	4.42

1-3	95	2.90	2.39	3.41

4-7	48	2.84	2.36	3.31

8-18	42	3.81	1.42	6.19

p = 0.630 with One-way ANOVA				

**Readmission Length of Stay For Neck Abscess Patients**				

**Age ** **(years)**	**N**	**Mean**	**Lower 95**%** CL for Mean**	**Upper 95**%** CL for Mean**

<1	6	3.37	0.43	6.31

1-3	11	1.91	0.53	3.29

4-7	5	1.92	0.49	3.35

8-18	5	0.93	0.56	2.43

p = 0.268 with One-way ANOVA				

**Table 2:** Percentage of children in each age group who underwent operative intervention for neck abscess with location of abscess (% represents % for each cell).

**Age (years)**	**Frequency (N)**	**(**%** of Age Group Treated Surgically)**	**Lateral Neck **(%)	**Retropharyngeal **(%)	**Parapharyngeal **(%)	**Other **(%)
<1	44	86.3	33 (89.2)	7 (77.8)	3 (100.0)	1 (50.0)

1-3	73	76.8	48 (84.2)	15 (65.2)	9 (61.5)	1 (100.0)

4-7	30	66.7	16 (100)	10 (47.6)	4 (57.1)	1 (100.0)

8-18	18	51.4	9 (64.3)	6 (54.5)	3 (60.0)	4 (0)

p = 0.004 with Kruskal-Wallis test for age distribution.

**Table 3:** Presenting symptoms and signs of pediatric patients diagnosed with neck abscess for each age group.

**Age (years)**	**Fever **(%)	**Swelling **(%)	**Neck Pain**	**Cervical Lymphadenopathy **(%)	**Neck Stiffness **(%)	**Sore Throat **(%)	**Dysphagia **(%)	**Cough **(%)	**Airway Obstruction **(%)	**Vomiting **(%)	**Dysphonia **(%)	**Diarrhea **(%)
**<1**	77.6	95.9	60	63.2	12.5	14.3	41.2	29.4	36.8	6.7	0	7.7

**1-3**	86.5	92.0	75.5	84.4	45.0	44.4	37.5	32.4	21.4	33.3	0	4.0

**4-7**	95.6	86.1	100.0	81.5	88.5	83.3	57.9	38.9	28.6	31.3	21.4	18.8

**8-18**	77.4	84.8	90.3	75.0	68.4	64.0	56.0	7.1	30.8	31.3	13.3	0

**N for which data is available**	214	193	143	107	101	99	93	83	74	77	67	67

**p value**	0.104	0.411	0.001_ _^*∗*^	0.116	<0.001_ _^*∗*^	0.001_ _^*∗*^	0.426	0.336	0.843	0.038	0.035	0.350

^*∗*^Significant difference (p < 0.05); Kruskal-Wallis test with Bonferroni correction.

These percentages are for cases where the symptom was noted in the medical record as present or absent. If there was no mention of the symptom, the chart was excluded, resulting in higher overall percentages.

**Table 4:** Location of abscess.

**Location**	**Frequency**	%	**Age < 1 yr (**%**)**	**Age 1-3 yrs (**%**)**	**Age 4-7 yrs (**%**)**	**Age 8 -18 yrs (**%**)**
**Lateral neck**	126	51.0	72.5	60.4	35.6	40.0

**Retropharyngeal**	62	25.1	5.9	24.0	44.4	28.6

**Parapharyngeal**	25	10.1	17.6	13.5	8.9	14.3

**Combination of Retropharyngeal and Parapharyngeal**	7	2.8	0	1.1	8.9	5.7

**Other **	8	3.2	3.9	0	2.22	11.4

Note that 15 patients did not have a specific location beyond neck defined in the medical record.

**Table 5:** Findings for computed tomography (CT) scan. 43 patients had multiple findings.

**CT Results (N = 195)**	**Frequency**	%	**Age < 1 yr **(%)	**Age 1-3 yrs **(%)	**Age 4-7 yrs **(%)	**Age 8 -18 yrs **(%)
**Hypodense collection**	95	48.7	42.5	51.9	48.8	48.6

**Rim enhancing lesion**	51	26.2	30.0	22.8	41.5	11.4

**Lymphadenopathy/cervical adenitis**	24	12.3	15	8.9	7.3	14.3

**Loculated lesion**	27	13.8	2.5	6.3	7.3	2.9

**Sinusitis**	4	2.1	2.5	2.5	2.4	0

**Jugular thrombus**	3	1.5	0	3.8	0	0

**Parotitis**	2	1.0	2.5	0	2.4	0

**Mastoiditis**	1	0.5	0	0	0	2.9

**Mediastinitis**	1	0.5	0	1.3	0	0

**Table 6:** Odds ratios and 95% confidence intervals.

**Category**	**Odds Ratio**	**95**%** CI Lower Limit**	**95**%** CI Upper Limit**	**P-Value**
**Child < 1 yr old (infant) undergoing surgery**	2.38	1.10	5.19	0.029_ _^*∗*^

**Child with lateral neck site undergoing surgery**	4.12	2.14	7.93	<0.001_ _^*∗*^

**Child having at least 1 cm radiolucency on CT undergoing surgery**	4.82	1.29	18.02	<0.001_ _^*∗*^

**Infant having a lateral neck site**	2.66	1.30	5.47	0.008_ _^*∗*^

**Infant having at least 1 cm radiolucency on CT**	0.63	0.15	2.55	0.514

**Infant having at least 2 cm radiolucency on CT**	1.07	0.47	2.44	0.865

**Infant with lateral neck site undergoing surgery**	2.275	0.89	5.82	0.086

**Infant with at least 1 cm radiolucency undergoing surgery**	3.00	1.01	8.94	0.048_ _^*∗*^

**Infant with lateral neck site and at least 1 cm radiolucency undergoing surgery**	2.49	0.74	8.43	0.142

*∗* denotes significantly different at P = 0.05, Mantel-Haenszel statistic.

## References

[1] Cabrera C. E., Deutsch E. S., Eppes S. (2007). Increased incidence of head and neck abscesses in children. *Otolaryngology—Head and Neck Surgery*.

[2] Craig F. W., Schunk J. E. (2003). Retropharyngeal abscess in children: clinical presentation, utility of imaging, and current management. *Pediatrics*.

[3] Freling N., Roele E., Schaefer-Prokop C., Fokkens W. (2009). Prediction of deep neck abscesses by contrast-enhanced computerized tomography in 76 clinically suspect consecutive patients. *The Laryngoscope*.

[4] Shah S., Sharieff G. Q. (2007). Pediatric Respiratory Infections. *Emergency Medicine Clinics of North America*.

[5] Raffaldi I., Le Serre D., Garazzino S. (2015). Diagnosis and management of deep neck infections in children: The experience of an Italian paediatric centre. *Journal of Infection and Chemotherapy*.

[6] Abdel-Haq N., Quezada M., Asmar B. I. (2012). Retropharyngeal abscess in children: The rising incidence of methicillin-resistant staphylococcus aureus. *The Pediatric Infectious Disease Journal*.

[7] Ridder G. J., Technau-Ihling K., Sander A., Boedeker C. C. (2005). Spectrum and management of deep neck space infections: An 8-year experience of 234 cases. *Otolaryngology—Head and Neck Surgery*.

[8] Long S. S., Pickering L., Prober C. G. (2012). Infections Related to the Upper and Middle Airways. *Principles and Practice of Pediatric Infectious Diseases*.

[9] Philpott C. M., Selvadurai D., Banerjee A. R. (2004). Paediatric retropharyngeal abscess. *The Journal of Laryngology & Otology*.

[10] Chi T.-H., Tsao Y.-H., Yuan C.-H. (2014). Influences of patient age on deep neck infection: Clinical etiology and treatment outcome. *Otolaryngology - Head and Neck Surgery*.

[11] Page N. C., Bauer E. M., Lieu J. E. C. (2008). Clinical features and treatment of retropharyngeal abscess in children. *Otolaryngology—Head and Neck Surgery*.

[12] McClay J. E., Murray A. D., Booth T. (2003). Intravenous antibiotic therapy for deep neck abscesses defined by computed tomography. *Arch Otolaryngol Head Neck Surgery*.

[13] Wong D. K. C., Brown C., Mills N., Spielmann P., Neeff M. (2012). To drain or not to drain - Management of pediatric deep neck abscesses: A case-control study. *International Journal of Pediatric Otorhinolaryngology*.

[14] Jakubowski L. A., Shariat-Madar B., McCormick M. E., Sulman C. G., Chun R. H. (2015). Recurrence patterns of cervical infections in children. *International Journal of Pediatric Otorhinolaryngology*.

[15] Cmejrek R. C., Coticchia J. M., Arnold J. E. (2002). Presentation, diagnosis, and management of deep-neck abscesses in infants. *Archives of Otolaryngology—Head and Neck Surgery*.

[16] Friedman N. R., Mitchell R. B., Pereira K. D., Younis R. T., Lazar R. H. (1997). Peritonsillar abscess in early childhood: Presentation and management. *Archives of Otolaryngology—Head and Neck Surgery*.

[17] Ungkanont K., Yellon R. F., Weissman J. L., Casselbrant M. L., Gonzáalez-Valdepena H., Bluestone C. D. (1995). Head and neck space infections in infants and children. *Otolaryngology—Head and Neck Surgery*.

[18] Novis S. J., Pritchett C. V., Thorne M. C., Sun G. H. (2014). Pediatric deep space neck infections in U.S. children, 2000-2009. *International Journal of Pediatric Otorhinolaryngology*.

[19] Grisaru-Soen G., Komisar O., Aizenstein O., Soudack M., Schwartz D., Paret G. (2010). Retropharyngeal and parapharyngeal abscess in children-Epidemiology, clinical features and treatment. *International Journal of Pediatric Otorhinolaryngology*.

[20] Johnston D., Schmidt R., Barth P. (2009). Parapharyngeal and retropharyngeal infections in children: Argument for a trial of medical therapy and intraoral drainage for medical treatment failures. *International Journal of Pediatric Otorhinolaryngology*.

[21] Duggal P., Naseri I., Sobol S. E. (2011). The increased risk of community-acquired methicillin-resistant Staphylococcus aureus neck abscesses in young children. *The Laryngoscope*.

